# Indices of body fat distribution for assessment of lipodysthrophy in people living with HIV/AIDS

**DOI:** 10.1186/1756-0500-5-543

**Published:** 2012-10-02

**Authors:** Aline Francielle Mota Segatto, Ismael Forte Freitas Junior, Vanessa Ribeiro Dos Santos, Kelly Cristina de Lima Ramos Pinto Alves, Dulce Aparecida Barbosa, Alexandre Martins Portelinha Filho, Henrique Luiz Monteiro

**Affiliations:** 1 Department of Physiotherapy, Univ. Estadual Paulista, Campus of Presidente Prudente, São Paulo, Brazil; 2 Departamento de Educação Física, Universidade Estadual PaulistaUNESP, Rua Roberto Simonsen, 305 Centro Educacional, Presidente Prudente, São Paulo, CEP 19.060-900, Brasil; 3 Physical Education, Univ. Estadual Paulista, Campus of Rio Claro, São Paulo, Brazil; 4 Department of Nursing, São Paulo Federal University, São Paulo, Brazil; 5 Sexually Transmitted Diseases Center of São Paulo State, Presidente Prudente, São Paulo, Brazil; 6 Department of Physical Education, Univ. Estadual Paulista, Campus of Bauru, São Paulo, Brazil

**Keywords:** AIDS/HIV, Abdominal obesity, Lipodysthrophy, Body composition

## Abstract

**Background:**

Metabolic and morphological changes associated with excessive abdominal fat, after the introduction of Antiretroviral Therapy, increase the risk of cardiovascular disease in people living with HIV/AIDS(PLWHA). Accurate methods for body composition analysis are expensive and the use of anthropometric indices is an alternative. However the investigations about this subject in PLWHA are rare, making this research very important for clinical purpose and to advance scientific knowledge. The aim of this study is to correlate results of anthropometric indices of evaluation of body fat distribution with the results obtained by Dual-energy X-Ray Absorptiometry(DEXA), in people living with HIV/AIDS.

**Methods:**

The sample was of 67 PLWHA(39 male and 28 female), aged 43.6+7.9 years. Body mass index, conicity index, waist/hip ratio, waist/height ratio and waist/thigh were calculated. Separated by sex, each index/ratio was plotted in a scatter chart with linear regression fit and their respective Pearson correlation coefficients. Analyses were performed using Prism statistical program and significance was set at 5%.

**Results:**

The waist/height ratio presented the highest correlation coefficient, for both male (r=0.80, p<0.001) and female (r=0.87, p <001), while the lowest were in the waist/thigh also for both: male group (r=0.58, p<0.001) and female group (r=0.03, p=0.86). The other indices also showed significant positive correlation with DEXA.

**Conclusion:**

Anthropometric indices, especially waist/height ratio may be a good alternative way to be used for evaluating the distribution of fat in the abdominal region of adults living with HIV/ADIS.

## Background

With the onset of the treatment with antiretroviral therapy (HAART) in the 90s, there was a significant increase in survival and quality of life of people living with HIV/AIDS (PLWHA), due to the action of this therapy in the fight against infectious and opportunist diseases [1]. However, pharmacological control of the disease has the side effect of altering body fat distribution, characterized by morphological changes (lipodystrophy) that result in excess fat in the central region of the body that increases the risk of cardiovascular diseases [2]. Despite some patients without HAART treatment present lypodistrophy, most of studies indicate that lypodistrophy in those under HAART, are time-dependent [3,4].

Methods of analysis of body composition such as Dual Energy X-Ray Absorptiometry (DEXA) and Computed Tomography are considered the accurate techniques for body composition assessment for PLWHA [5,6]. However, the acquisition of sophisticated equipments or even the individual cost of each exam has made it difficult the access to most population [7].

Given the difficult access to these techniques, it becomes primordial the use of procedures for the diagnosis of abdominal obesity in PLWHA. The anthropometric indices can be used as simple procedures to classify PLWHA on the risk of diseases related to excess central obesity, as they are widely used successfully for this purpose in non HIV-infected adult population [8].

However, investigations concerning the use of anthropometric indices to assess the risks related to excess fat in PLWHA are still scarce, and literature suggests that, for most methods of body composition assessment, the parameters assumed to be true for one population group may not be the same for others [9].

The objective of the present study is to correlate results of some anthropometric indices with values obtained by DEXA, in order to assess whether these indices can be used for diagnosis of excess central fat in people living with HIV/AIDS.

## Methods

Cross-sectional descriptive study with people living with HIV/AIDS attended by Sexually Transmitted Diseases Center of São Paulo State (STDC) was conducted in the city of Presidente Prudente, São Paulo, Brazil. In 2009 there were a total of 653 patients attended in the STDC, which is a governmental health service that provides diagnostic and treatment of sexually transmitted diseases. Between May to July, a total of 286 patients, aged 18 or over, were attended and all of them were invited to participate in the present study. From this amount, 131 accepted to participate and attended all inclusion criteria of the present study.

The inclusion criteria were: (1) age 18 years or over; (2) to be patient of the Sexually Transmitted Diseases Center of São Paulo State, for, et least, one year; (2) to be treated with the antiretroviral therapy for, at least, one year. Those who made use of a pacemaker, anti-hyperlipidemic drugs, pregnant women and prisoners were not included.

Thus, the final sample was composed by 67 individuals aged 44.0 ± 7.6 years (39 male, 45.0 ± 8.4 years and 28 female, 42.0 ± 6.3 years).

Evaluations were performed by researches with experience in assessing body composition.at the Centro de Estudos e Laboratório de Avaliação e Prescrição de Atividades Motoras (CELAPAM) da Universidade Estadual Paulista “Julio de Mesquita Filho” FCT-Unesp Presidente Prudente.

### Procedures

Body weight (kg), height (m) and waist, hip and thigh (cm) circumferences were recorded to estimate the suggested indices.

The weight was measured on a digital electronic scale (Filizzola PL 150, Filizzola Ltda) to the nearest 0.1 kg. Height was measured using a stadiometer to the nearest 0.1 cm and 2 meters extension. The subjects were wearing light clothing and without shoes. The waist, hip and thigh circumferences were performed using a metal measuring tape, Sanny, accurate to 0.1 cm and maximum length of 2 m. The measurement of waist circumference was performed with the tape positioned at the smallest circumference between the iliac crest and last rib. The hip circumference was measured with the measuring tape positioned at the largest circumference at the gluteus maximus.

All anthropometric measurements followed procedures described by Lohman [10] and Freitas Jr. et al. [11] and were collected by trained staff.

### Dual Energy X-ray Absorptiometry (DEXA)

Whole body composition was measured with a Lunar DPX-NT scanner (GE Medical, Software: Lunar DPX Encore 2007 version 11.40.004, Madison, WI).

All measurements were made at the CELAPAM with constantly controlled temperature. Each morning before any measurements were taken, the device was calibrated by the same researcher and, according to the reference values provided by the manufacturer, the tests presented high reliability.

The subjects were scanned wearing light clothing while lying flat on their back with arms by the side, without moving during the measurement. The equipment provided measurements of percentage of the trunk fat mass (visceral plus subcutaneous).

### Index and ratios

The following indices and ratios were calculated:

Body Mass Index:

$$\frac{\text{weight}\left(\text{kilos}\right)}{\text{height}\left(\text{meters}\right)}$$

Conicity index:

waist circumference (meters)

$$0,109\times \sqrt{\frac{\text{Body}\;\text{Weight}\left(\text{kilos}\right)}{\text{Height}\left(\text{meters}\right)}}$$

Waist/hip ratio:

$$\frac{\text{waist}\;\text{circumference}\left(\text{centimeters}\right)}{\text{hip}\;\text{circumference}\left(\text{centimeters}\right)}$$

Waist/height ratio:

$$\frac{\text{waist}\;\text{circumference}\left(\text{centimeters}\right)}{\text{Height}\;\text{centimeters}}$$

Waist/thigh ratio:

$$\frac{\text{waist}\;\text{circumference}\left(\text{centimeters}\right)}{\text{thigh}\;\text{circumference}\left(\text{centimeters}\right)}$$

The study was approved by the Ethics Committee on Human Experimentation of the Sao Paulo State University at Presidente Prudente. (protocol number 2101/08) and all research participants gave written informed consent after receiving a thorough explanation of the research project.

### Statisitical analysis

For data analysis, the sample was divided by sex, whereas body composition is different between men and women.

The normality of the analyzed data was tested by the test of Komolgorov-Smirnov (K-S).

Mean, standard deviation and the Pearson product–moment correlation coefficient(r) were calculated in SPSS version 13.00 (SPSS Inc., Chicago, IL).

The sample was split by sex and each index/ratio was plotted in the scatter plot with linear regression. In the presentation of each figure the values of correlation and their significance have been described. The graphics were performed in the statistical program Prism and the Statistical significance (p) was set at 5%.

## Results

The general characteristics of the sample are described in Table 1.

**Table 1:** Mean values and standard deviation of anthropometric variables according to sex

**Variables**	**Male**	**Female**
Age (years)	44.7 ± 8.4	42.3 ± 7.3
Weight (Kg)	71.6 ± 13.74	63.6 ± 12.4
Height (cm)	170.9 ± 7.34	159.0 ± 5.64
Waist Circumference (cm)	88.9 ± 12.75	82.6 ± 12.25
Thigh Circumference (cm)	48.4 ± 4.30	51.5 ± 8.19
Hip Circumference (cm)	94.4 ± 8.38	97.4 ± 10.37
Trunk length (cm)	90.4 ± 3.48	84.8 ± 2.59
Body mass index (Kg/m^2^)	23.5 ± 4.62	23.8 ± 5.63
Conicity index (cm/Kg/m)	1.3 ± 0.09	1.2 ± 0.07
Waist hip ratio (cm/cm)	0.9 ± 0.08	0.9 ± 0.09
Waist estature ratio (cm/cm)	0.5 ± 0.08	0.5 ± 0.07
**Waist thigh ratio (cm/cm)**	1.8 ± 0.20	1.6 ± 0.32
Trunk fat mass (kg)	11.3 ± 5.4	10.0 ± 5.5

**Figure 1:**
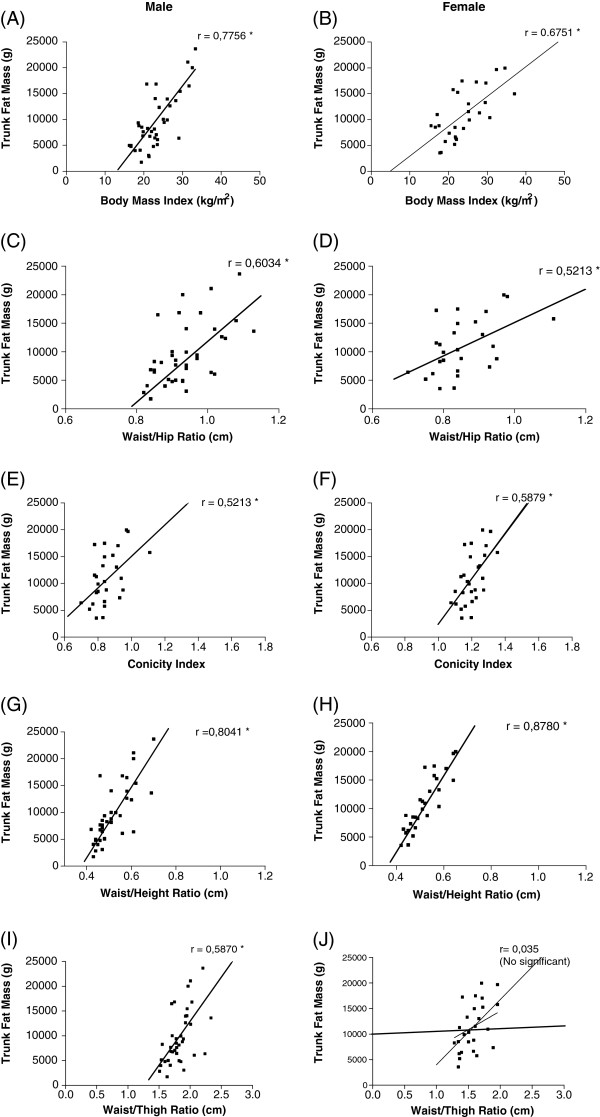
Scatterplots comparing values of trunk fat mass (grams) and its Indices / Ratio for sex. (Attached separately).

In the results of Table 2 can be observed that all variables presented significantly correlation between index/ratios with trunk fat mass estimated by DEXA, except the waist/height ratio in the female group. In Figure 1, individual data for each correlation, for both sexes, can be better visualized.

**Table 2:** Mean values, standard deviation and correlation coefficient between indices/ratios with trunk fat mass estimated by DEXA

**Variables**	**Sex**	**Mean ± SD**	**r**	**p**
Body mass índex (Kg/m^2^)	M	23.46 ± 4.62	0.77	0.000
	F	23.83 ± 5.63	0.67	0.000
Conicity index (cm/Kg/m)	M	1.26 ± 0.08	0.52	0.000
	F	1.20 ± 0.06	0.58	0.000
Waist/hip ratio (cm/cm)	M	0.94 ± 0.07	0.60	0.000
	F	0.85 ± 0.09	0.52	0.004
Waist/ height ratio (cm/cm)	M	0.52 ± 0.08	0.80	0.000
	F	0.52 ± 0.06	0.87	0.000
Waist/thigh ratio (cm/cm)	M	1.83 ± 0.19	0.58	0.000
	F	1.63 ± 0.32	0.03	0.859
Trunk fat mass (kg)	M	10.39 ± 5.96	-	-
	F	10.90 ± 4.58	-	-

M = male, F = female, SD = Standard Deviation.

## Discussion

The introduction of drugs against HIV is considered a milestone in the history of the disease, because it increased, significantly, survival and quality of life of people living with HIV/AIDS [12-14]. Despite the benefits with the treatment, side effects, as lipodystrophy, deserve attention due to health damages [15].

The present study investigated the correlation between anthropometric indices and the trunk fat mass measured by a more accurate technique.

National [16,17] and international [18,19] studies make use of indices to classify the nutritional status or to relate excess weight and cardiovascular risk, but little is investigated about the use of such methods in PLWHA.

Despite being a body fat indicator more related to total body fat than to the central fat, and be limited to indicate body fat with risk cardiovascular risk [20,21], the body mass index (BMI), in the present study, showed high correlation with the trunk fat mass in both sexes.

The high correlation between BMI and total body mass observed in healthy adults [22,23], is one of the limitations that do not allow differentiating excess weight caused by fat or lean fat mass [24,25]. However, the specificity of the body composition of the investigated group, as the excessive accumulation of fat in the trunk region, known as lipohypertrophy or the little amount of fat in the limb areas, known as lipoatrophy [26,27], should be responsible for the high rates of correlation between BMI and trunk fat mass. No similar studies were found that can permit a deep discussion of the results.

From the studies found in the literature, that used the other body indices to evaluate excess of central fat in PLWHA, only two [3,28] used waist/hip ratio.

The waist/hip ratio results in this study corroborate the findings of Florindo *et al.*[3] and Padilla *et al.*[28] in studies performed in adult PLWHA, Spanish and Brazilian, respectively. They also found a positive correlation between waist/hip ratio with abdominal fat estimated by gold standard equipment (computed tomography). These authors inferred that, this may be a good method to estimate abdominal fat in PLWHA.

The waist/thigh ratio (WTR) was proposed as an alternative to waist/hip ratio (WHR) to estimate body fat distribution and the prediction of morbidities, by using the circumference of the thigh, which is unaffected by variations in the pelvic architecture, like happens with the hip [29]. However, in this study, the highest correlation values were observed between WHR and DEXA, while the WTR showed no significant correlation for females. Similar results were also observed by Vasques *et al.*[30], who studied non infected adult subjects. It is possible that, the low correlation values of WTR are results of lipohypertrophy, lipoatrophy or both, resulting in variations in the morphological distribution of body fat and reflect on the WTR. Our results suggest that this index needs to be further studied for use as a risk factor in PLWHA.

The conicity index is based on the proposition that people who accumulate fat in the abdominal area have a body shape like a double cone, two cones with common base, arranged one over the other [31]. In the present investigation, the conicity index showed a better correlation with DEXA value than the WTR for females. According to Guedes and Guedes [32] the main advantage of the conicity index compared with WTR is to present higher sensitivity for analysis of body fat distribution in non infected subjects, considering the probable joint variation of the measures of waist and hip circumference.

The index that presented the highest correlation with trunk body fat measured by DEXA was a waist/height ratio (WHeR), with r = 0.80 for males and 0.87 for females. Lin *et al*. [33] studied non infected adult individuals and obtained results consistent with our study and found strong association with excess of abdominal fat and with several cardiovascular risk factors. The advantage in applying this indicator is related to the adjustment for height that might facilitate direct comparisons between different populations [34]. For PLWHA, another positive aspect to this technique is that its ratio use only of the height and waist variables, focusing thus in the central fat distribution that relates to cardiac risk factors, without suffering the influence of variables of body composition in peripheral segments of the body that can be altered by the lipodystrophy syndrome and, thus, interfere the results.

Despite the importance of the findings of this study, some limitations should be mentioned, like the fact that the period of time that the subjects were infected with the virus was not investigated and also did not provide information about the presence or absence of limb lipoatrophy and trunk lipohypertrophy.

Despite the significant results found in the present study, some limitations need to be stated. The two main limitations are that, the small sample size limit the extrapolation of the results and the cross sectional study do not permit to establish a cause-effect effect of the all index/ratio and cardiovascular risk in PLWHA.

## Conclusions

In conclusion, our results suggest that, the use of anthropometric indices can be useful for assessing central fat distribution of PLWHA for research purpose and in monitoring health services that do not have more accurate and sophisticated equipment to assess body fat distribution. However, the choice of the method should be careful and further studies are necessary to confirm our results.

## Competing interests

The authors declare that they have no competing interests.

## Authors’ contributions

AFMS: Main mentor and responsible for DEXA assessment of patients, organization of the data and redaction of the methods of the manuscript. IFFJ: supervisor of the manuscript and responsible for the statistical analysis. VRS: Responsible for anthropometrical and DEXA assessment of patients, organization of the data and redaction of the methods of the manuscript. KCLRPA: Responsible for contacting the patients, collection of blood samples, and participated in the discussion of the manuscript. DAB: Responsible for supervising the data collection, interpretation of the results and redaction of the manuscript. AMPF: Responsible for the clinical assessment of the patients and interpretation of the results. HLM: Responsible for the statistical analysis and had a substantial contribution in the discussion of the results of the manuscript. All authors read and approved the final manuscript.
